# Effects of several UV-protective substances on the persistence of the insecticidal activity of the Alphabaculovirus of *Chrysodeixis chalcites* (ChchNPV-TF1) on banana (*Musa acuminata*, Musaceae, Colla) under laboratory and open-field conditions

**DOI:** 10.1371/journal.pone.0250217

**Published:** 2021-05-12

**Authors:** Taylan Çakmak, Oihane Simón, Mehmet Bora Kaydan, Denis Achiri Tange, Agueda Mª. González Rodríguez, Ana Piedra-Buena Díaz, Primitivo Caballero Murillo, Estrella Hernández Suárez

**Affiliations:** 1 Unidad de Protección Vegetal, Instituto Canario de Investigaciones Agrarias (ICIA), Valle de Guerra, Tenerife, Spain; 2 Bioinsecticidas Microbianos, Institute for Multidisciplinary Research in Applied Biology (IMAB), Universidad Pública de Navarra (UPNa), Mutilva, Navarra, Spain; 3 Biotechnology Application and Research Centre, Çukurova University, Adana, Turkey; 4 İmamoğlu Vocational School, Çukurova University, Adana, Turkey; 5 Faculty of Agriculture, Department of Plant Protection Balcali, Çukurova University, Adana, Turkey; 6 Departamento de Botánica, Ecología y Fisiología Vegetal, Universidad de La Laguna, San Cristóbal de La Laguna, Tenerife, Spain; University of Carthage, TUNISIA

## Abstract

Alphabaculovirus of *Chrysodeixis chalcites* (ChchNPV-TF1) has been investigated as a useful bioinsecticide against *C*. *chalcites* (Esper) (Lepidoptera: Noctuidae) in banana crops. This study investigated the effects of several substances on the persistence of ChchNPV-TF1 under field conditions in the Canary Islands. Natural photoprotective substances, such as moringa, cacao, green tea, benzopurpurine, charcoal, iron dioxide, benzimidazole, kaolinite, and bentonite, were first evaluated under laboratory conditions using a Crosslinker as UV light source at 200 J/cm^2^. The photoprotective substances were divided into three groups: low protection (0–8%; kaolinite), intermediate protection (48–62%; green tea, moringa, bentonite and cacao) and high protection (87–100%; charcoal, iron ioxide). Benzopurpurine and benzimidazole did not provide any photoprotective effects. Two of the substances that yielded the best results, 1% cacao and 1% charcoal, were selected for the open-field experiment in a banana plantation. The persistence of ChchNPV-TF1 OBs (occlusion bodies) on leaf surfaces with sunlight exposure was analysed by comparing the initial mortality of 2^nd^ instar *C*. *chalcites* larvae with the mortality observed at various intervals postapplication. The mortality rates decreased over time in all treatments and were always higher in the UV-protective substance-treated parcels. The 1% charcoal treatment exhibited the highest protection in both the laboratory and field experiments. No specific interference of UV-protective substances on the maximum photochemical efficiency of banana plants was observed under field conditions.

## Introduction

The Canary Islands is the largest producer of banana (*Musa acuminata* Colla) in Spain [1]. In 2017, the Canary Islands produced 437.782 tons of banana, which was worth 104.416 thousand euros, representing approximately 39% of the total agricultural production of the archipelago [1]. Most of this banana production is destined for markets in mainland Spain (91%), and only a small fraction is exported to Western Europe (0.1%), whereas the remainder (8.9%) is consumed locally [2].

The golden twin-spot moth, *Chrysodeixis chalcites* (Esper) (Lepidoptera: Noctuidae), is a highly polyphagous species that causes severe damage in a large number of cultivated plants in many regions of Europe, the Mediterranean, the Middle East and Africa [3, 4], and currently, this species is one of the most important pests affecting banana crops in the Canary Islands, both in mesh-built greenhouses and in open fields [5, 6]. The larvae of this moth feed on several fruit, horticultural, ornamental and forest crops, such as banana (*Musa* sp. L.), cotton (*Gossypium* L.), alfalfa (*Medicago sativa* L.), cabbage (*Brassica oleracea* L.), sunflower (*Helianthus annuus* L.), geranium (*Pelargonium* Charles L’Héritier), bean (*Phaseolus vulgaris* L.), corn (*Zea mays* L.), turnip (*Brassica rapa* L.), potato (*Solanum tuberosum* L.), cucumber (*Cucumis sativus* L.*)*, pepper (*Piper nigrum* L.*)*, soybean (*Glycine max* L.), tobacco (*Nicotiana tabacum* L.) and tomato (*Solanum lycopersicum* L.) [3].

Chemical insecticides have normally been employed to control this pest in the Canary Islands [6, 7]; however, the low number of active substances authorised by the European Union for use in banana limits the possible application of alternative active substances, thereby increasing the risks for development of insecticide resistance associated with this type of pest control measure [8]. In addition, chemical insecticides have considerable negative consequences for both farmers and consumers, as well as for beneficial insects. Insecticides that do not leave xenobiotic residues are needed to be compatible with integrated pest management (IPM), which seeks to conserve natural enemy populations in agroecosystems. Biological insecticides based on *Bacillus thuringiensis* var. kurstaki have shown to be effective in greenhouse and field crops against *C*. *chalcites* [6, 7], and the recent discovery of the *C*. *chalcites* nucleopolyhedrovirus (ChchNPV) has been an important advance in the control of this pest [9–11].

The *C*. *chalcites* nucleopolyhedrovirus (ChchNPV-TF1), which was isolated and characterized from banana farms in southern Tenerife, was investigated as an effective insecticide [9–11]. A formulation of this baculovirus for commercial purposes still requires further improvements for its successful application in IPM programmes. One of the factors that clearly disrupts baculovirus efficacy is solar ultraviolet (UV) radiation, which affects the persistence of baculovirus occlusion bodies (OBs) deposited on plant surfaces, rapidly reducing the efficacy of the baculovirus [10, 11]. An improved formulation of this baculovirus for commercial purposes is required for successful application in IPM programs.

In general, the efficacy of baculoviruses drastically decreases within the first 48 h after spraying as OBs are rapidly inactivated by solar UV radiation [12–16]. A number of substances providing UV protection to baculovirus formulations, including dyes, fluorescent brighteners, lignin derivatives, antioxidant or oxidative enzymes, such as dilodin, inol, vitamins, folic acid, riboflavin, and pyridoxine, have been tested to determine whether they protect entomopathogenic viruses from UV rays [17–20]. However, these substances were the so called optical brighteners, most of them from synthetic origin, expensive or difficult to apply in field. Consequently, the latest studies on photoprotectant substances evaluate natural derived antioxidants, mainly plant derived extracts [21–25].

Despite their “natural” origin, these substances might affect the photosynthetic activity of plants. The chlorophyll fluorescence technique has been widely used in the diagnosis of plant health for decades [26–28]. Many fluorescence parameters or indexes have been applied to study the effect of different environmental stresses (e.g., light, temperature and heavy metals). Among these parameters, the Fv/Fm ratio is the most well-known and is closely linked to photosynthetic activity, reflecting the maximum photochemical efficiency of photosystem II [29].

In this study, several substances that were previously found to have photoprotective capacity, such as green tea, moringa, cacao, kaoline, iron oxide, charcoal, bentonite, benzopurpurine and benzimidazole [21, 22, 25, 30–33], were tested for the first time to determine their photoprotective activity to ChchNPV-TF1, and the joint effect virus-UV protectant on *Chrysodeixis chalcites* survival and plant photosynthetic activity. In our first assay, natural photoprotective substances were evaluated under laboratory conditions, and then two of the substances yielding the best results were tested in the field. For the first time, possible plant effects associated with the use of these ingredients, measured as the maximum photochemical efficiency of banana plants under field conditions, were also determined.

## Materials and method

### Insects

The larvae of *C*. *chalcites* used in bioassays and field trials were obtained from a laboratory-reared *C*. *chalcites* colony originally founded by insects collected (09/07/2016) from Las Galletas (28° 01’ 52” N, 16° 39’ 32” W), Tenerife, Canary Islands, Spain,. The colony was maintained in the Instituto Canario de Investigaciones Agrarias (ICIA), Tenerife, at 25 ± 1°C, 60 ± 80% relative humidity and a photoperiod of 16:8 h (light:dark). Larvae of *C*. *chalcites* were reared on a semisynthetic diet based on cornflour, wheat germ and yeast, while adults were fed with a 10% v/v aqueous honey dilution [34].

### Virus strain

ChchNPV-TF1 was mass-produced by infecting series of 100 6^th^ instar *C*. *chalcites* at 1×10^8^ OBs/ml by the droplet feeding method [35]. Inoculated larvae were placed in 24-well tissue culture plates with a semisynthetic diet and incubated at 25°C. Larval mortality was measured daily. Dead larvae with polyhedrosis symptoms were collected and stored at -20°C. OB cadavers were homogenized in 50 ml sodium docecil suflate (SDS) at 0,1% (wt/vol) with the aid of mortar, filtered through Mullin and centrifuged at 3,800 *x g* for 5 min. The resulting OB pellet was resuspended in the same volumen as the pellet (≈10 ml) with sterile water, and the OB concentration was determined by counting triplicate samples using an improved Neubauer haemocytometer (Superior Marienfield, Laude-Koeningshofen, Germany) under phase contrast microscopy at 400× magnification. Purified OBs were stored at 4°C until use. The identity of these OBs was confirmed by restriction endonuclease analysis using *Bgl*II [5, 36].

### Determining the ChchNPV-TF1 concentration

To determine the OB concentration to apply against 2^nd^ instar *C*. *chalcites*, five different viral concentrations were examined. The density of viable OBs on the banana leaf surface was estimated by a calibration curve of the bioassay. To this end, a 5-fold dilution series was used as concentrations: 160, 800, 4000, 2x10^4^, and 1x10^5^ OBs/ml, or a water control, were applied to the banana leaves collected from the untreated part of the parcel using a compressed-air hand sprayer (DEA 2000, Italia). These concentration ranges were previously determined to kill between between 5 to 95% of the experimental insects for each instar [10]. All treatments included 0.1% (v/v) Agral (Syngenta Agro S.A., Madrid, Spain) as a wetter sticker. Thirty minutes after application, when banana leaf plants were completely dry, 5-cm diameter banana leaf discs were carefully cut and placed into Petri dishes. Each Petri dish was infested with 30 2^nd^ instar *C*. *chalcites*. As a control, 30 larvae from the laboratory colony fed untreated banana discs. Larvae were individualized in 25-ml plastic cups with artificial diet 24 h postinfection and under controlled conditions at 25 ± 1°C, 70 ± 5% humidity, and a 16:8 h (light:dark) photoperiod. Larvae were inspected daily until death or pupation. The entire process was repeated five times.

### Determination of photoprotective activity of several substances under laboratory conditions

In Table 1, the photoprotective activity of several substances is shown. All these substances were assayed under laboratory conditions by being sprayed along with the virus onto banana leaves. A virus suspension was applied at 105 OBs/ml, which was expected to produce up to 80–100% mortality. The photoprotective substances were first applied at 10% dilutions; however, the substances remained on the banana leaves in a thick layer that might inhibit photosynthesis metabolism by acting as a physical barrier. Therefore, all substances were prepared at 1% dilutions.

**Table 1:** Natural substances that were assayed as UV-protectives under laboratory conditions.

Substance	Original Activity Remaining (OAR* %) (compared with control)	Company	Reference
Moringa	1%; 93%	Art. N° 55. Moringa Powder. Kräuterhaus Snct Bernhard (Helfensteinstr. 47, Bad Ditzenbach, Germany)	[21]
Cacao	10%; 50% 1%; 96% 1%; 85–100%	Cacao dream. O.K. Eco Su Alternativa Natural. Becerril de la Sierra (Madrid, Spain)	[22] [25] [25]
Green tea	1%; 85–100% 1%; 66% 1%; 100%	Art. No° 15. Green Tea Japan Sencha. Kräuterhaus Snct Bernhard (Helfensteinstr. 47, Bad Ditzenbach, Germany)	[25] [22] [22]
Benzopurpurine 4B	1%; 80% 1%; 75%	Art N° J64901. Alfa Aesar (Karlsruhe, Germany)	[17]
Charcoal	1%; 75% 5% 50%	Activated charcoal DARCO^®^. Art. N° 242276–250 g. Sigma-Aldrich (St. Louis, MO, USA).	[17] [37] [38, 39]
Benzimidazole	High protection	Art N° 194123–100 g. Sigma-Aldrich (St. Louis, MO, USA)	[37]
Kaolinite	1%; 80%	Art. N° 2180. Mezcla Perfecta (C/ Hermanos Álvarez Quintero 6, Madrid, Spain)	[31]
Bentonite	1% 96%	Art. N° 7222. Mezcla Perfecta (C/ Hermanos Álvarez Quintero 6, Madrid, Spain)	[30, 33]
Iron oxide	1-4mg/ml (UV 1/6-1/18)	Art. N° 12507. Mezcla Perfecta (C/ Hermanos Álvarez Quintero 6, Madrid, Spain)	[33]

A positive control, which contained only the virus without photoprotective substance, and a negative control, the water-only solution, were included in the analysis. These suspensions were sprayed onto banana discs. After waiting 30 min to allow the leaves to dry, they were placed in a cross-linker at 200 J/cm^2^ (Stratalinker Stratagene 1800 UV Crosslinker). Additionally, banana leaves that were sprayed with the virus only were included in the analysis, which might serve to determine if crosslinker irradiation worked well. Finally, nonirradiated discs sprayed with water only were also included as a negative control. A foliar surfactant 0.1% (v/v) Agral (Syngenta Agro S.A., Madrid, Spain) was included in each suspension as a wetter sticker. Banana discs of each treatment were placed in Petri dishes and infested with 30 larvae. Twenty-four hours later, larvae were individualized in 24-well Petri dishes containing an artificial diet. After one week, virus-induced mortality was evaluated. A total of 3 replicates were employed.

### Determination of the UV protection efficacy of 1% cacao and 1% charcoal under field conditions

Field experiments were performed under open air conditions during summer over two consecutive years, extending from 19/06/17 to 19/07/17 and from 27/09/18 to 11/10/18, coinciding with strong solar radiation. The air temperature (°C), photosynthetically active radiation (PAR) (Minikin QTi, EMS, Brno, CZ) and air humidity (%) (Minikin RTHi, EMS, Brno, CZ) were monitored 1.5 m above ground at the edge of the experimental field plot in both years during the course of the experiments. Internal data loggers continuously recorded 30-min averages of all measurements taken every 5 min.

An experimental banana plantation of approximately 700 m^2^ at the ICIA was selected for these trials and planted with young banana plants (Grand Nain cultivar). The experimental design consisted of randomized plots with four replicates per treatment. Experimental plots consisted of three banana plants surrounding a central plant; each plant had an average number of seven leaves each and was spaced 1 m from the closest plant.

Plots were sprayed with a negative control composed of water and surfactant only, a positive control that consisted of ChchNPV-TF1 at 10^9^ OBs/l (10^12^ OBs/ha), a suspension that included ChchNPV-TF1 at 10^9^ OBs/l plus 1% charcoal (charcoal + ChchNPV), and a suspension that included ChchNPV-TF1 at 10^9^ OBs/l plus 1% cacao (cacao + ChchNPV). Agral (Agro S.A., Madrid, Spain) wetter stickers were included in each suspension. These suspensions were sprayed directly onto banana plants and were exposed to natural solar radiation. Foliar sprays (1 litre per plant) were applied with a 16-litre backpack sprayer with 12 bar of pressure equipped with an extension lance and a full cone nozzle between 8.00 and 11.00 AM.

The effect of cacao and charcoal on the persistence of ChchNPV-TF1 on banana plants was measured indirectly by the mortality induced on 2^nd^ instar *C*. *chalcites* larvae fed the treated banana leaves at 1 h, 24 h, 72 h, 120 h and 168 h postapplication. To that end, at each postapplication moment, a 5-cm-diameter banana leaf disc from the central plant of each plot (three leaf discs by treatment) was carefully cut and placed into Petri dishes in the field and then brought to the laboratory and stored at 4°C until the bioassay was performed. Thirty to forty hungry 2^nd^ instar *C*. *chalcites* were sealed on these banana leaf discs (and therefore exposed to the residues of ChchNPV-TF1) for 24 h; thereafter, all larvae were individualized in 24-well Petri dishes containing artificial diets and maintained in a laboratory rearing chamber at 25 ± 1°C and a 16:8-h (L:D) photoperiod until death or pupation. Larvae were monitored daily, and those dead with the typical signs of polyhedrosis disease were individually frozen at -80°C for subsequent analysis. Larval mortality caused by the different virus formulations with protectants was compared with that produced by nonprotected virus treatment at each time point.

### Influence of cacao and charcoal on the photosynthetic activity of banana plants

The chlorophyll fluorescence was measured at midday in the middle section of sun-exposed leaves at 1, 3, 5, 7 and 14 days postapplication in the 2018 trial. The measurements were made with a portable fluorimeter (Handy PEA, Plant Efficiency Analyser, Hansatech, UK) on dark-adapted leaves (for 30 min) to determine basal fluorescence (Fo). After saturating the red light pulse (650 nm, 3000 μmol photons m^–2^ s^–1^) flashed by an array of ultrabright red light emitting diodes, the maximum fluorescence (Fm) was determined. From these parameters, the maximum photochemical efficiency (Fv/Fm) was calculated as the ratio (Fm-Fo)/Fm according to [28].

### Statistical analysis

To determine the optimal concentration, probit linear regression (SPSS. Ver 23) was used to estimate the concentration that kills 50% of test animals (LC50). Percentage mortality for laboratory and maximum photochemical efficiency were calculated for each treatment and subject to analysis of variance (ANOVA), as data were normally distributed. The significance between treatments for each concentration was determined by between-subject comparisons among the estimated means with Tukey’s HSD test (*P ≤* 0.05). A GLM repeated measure ANOVA was performed to evaluate the effect on larval mortality over time in the field trials. All analyses were performed using SPSS (IBM SPSS Statistics v. 23).

## Results

### Optimal dose of ChchSNPV-TF1 OBs against *C. chalcites*

**Fig 1:**
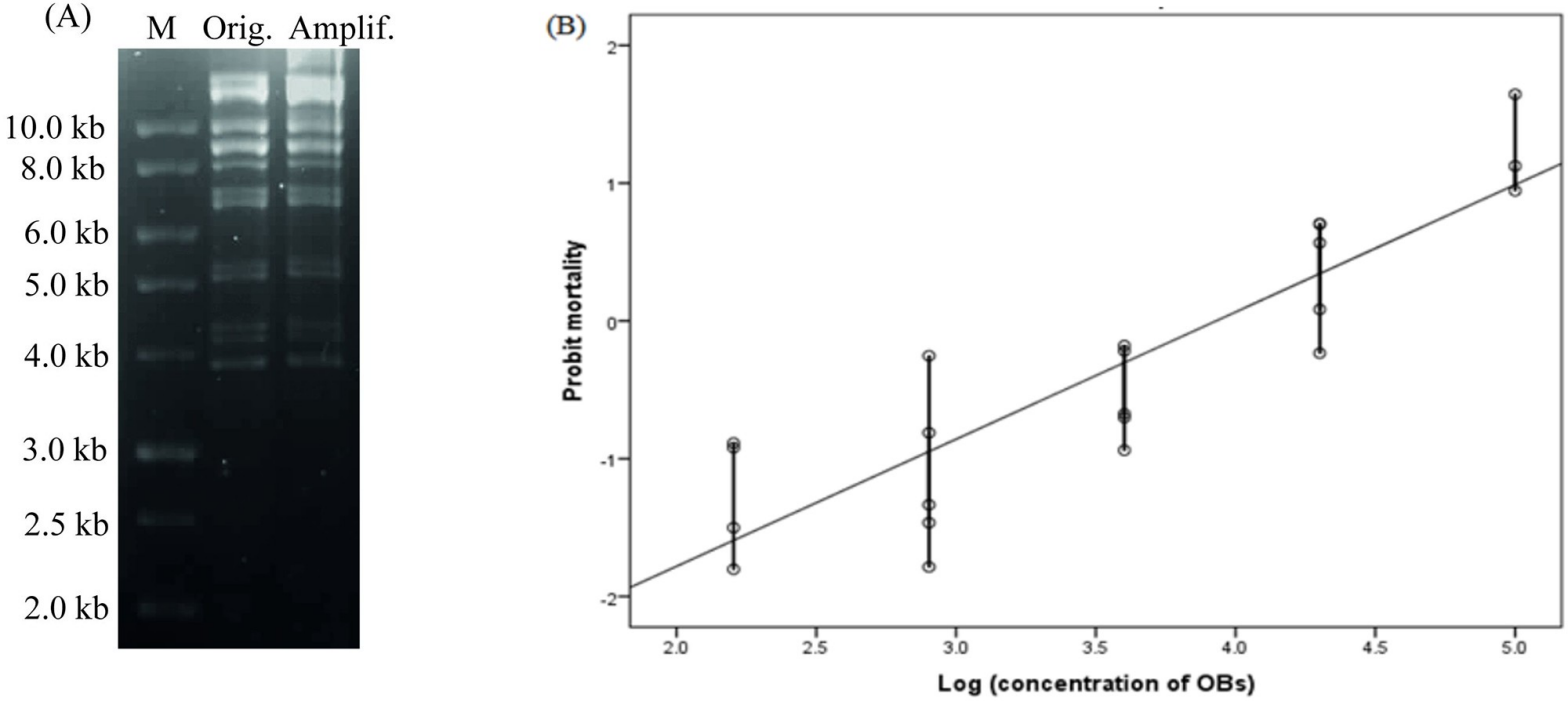
Restriction endonuclease analysis (*Bgl*II) of ChchSNPV-TF1 and probit regression line. (A) Restriction endonuclease analysis (*Bgl*II) following digestion of genomic DNA of the original inoculum (Orig.) and the laboratory-amplified (Amplif.) OBs of ChchSNPV-TF1. The DNA 1 kbMarker Ladder (Nippon, Tokyo, Japan) was used as a molecular size marker (M). (B) Probit regression line obtained after laboratory production was determined by bioassay in 2^nd^-instar *Chrysodeixis chalcites* larvae (y = 0.92x—3.63, R^2^ = 0.78).

The *Bgl*II profile of the baculovirus produced in laboratory-infected larvae was identical to that described previously [9, 40] (Fig 1A), which confirmed the identity and absence of cross-contamination during laboratory production of OBs. Furthermore, the LC_50_ of laboratory-produced ChchNPV-TF1 OBs for 2^nd^ instars of *C*. *chalcites* was 8.47 x 10^3^ OBs/ml (Fig 1B). This value is relatively similar to that of [10]. Data analysis demonstrated that there was a significant difference in larval mortality (F_5,24_ = 46.586, *P* < 0.0001).

### Photoprotective activity of several substances under laboratory conditions

**Fig 2:**
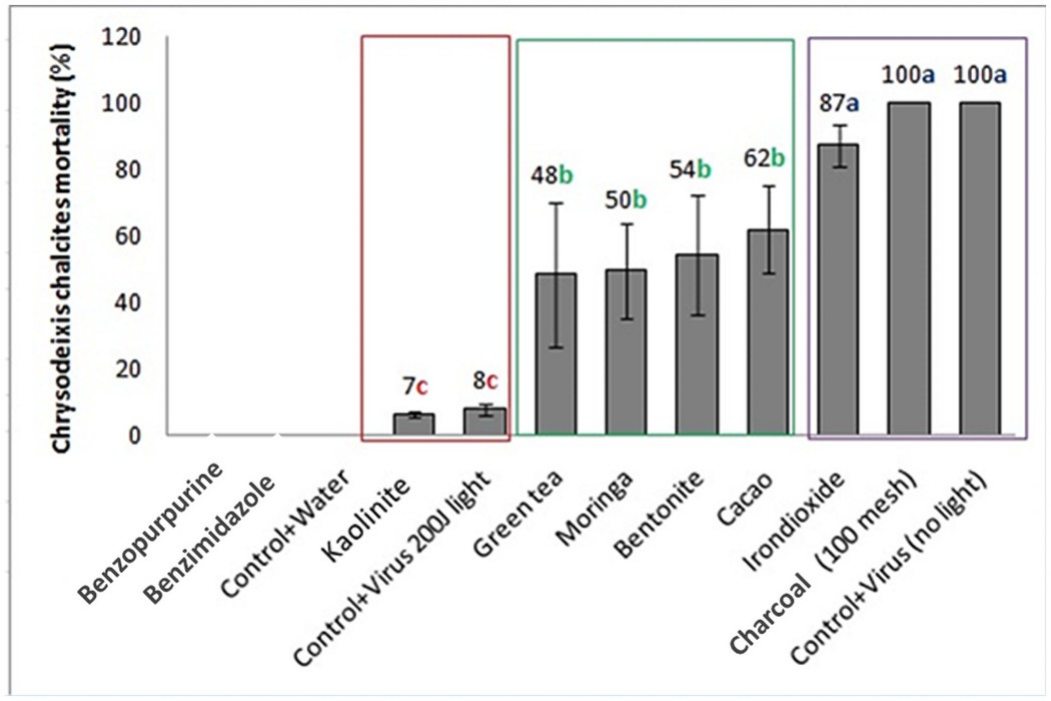
Percentage of virus-induced mortality of *C*. *chalcites* with selected UV-protective substances after exposure to 200J/cm2 radiation at cross-linker.

Nine different natural UV-protectant substances were evaluated under laboratory conditions (Fig 2). There was a significant difference between the treatments (F_11,24_ = 11.56. *P* < .0001). It was shown that three main groups were obtained by means of baculovirus-induced mortality on 2^nd^ instar *C*. *chalcites* larvae. It was determined that the group that presented high photoprotection, obtaining mortalities similar to the nonradiated virus, was charcoal and iron oxide, which produced mortality as high as 87–100% at cross-linker. The substances that presented intermediate photoprotection were cacao, green tea, moringa and bentonite. The mortality of *C*. *chalcites* from this second group ranged from 48–62%. Finally, there was a group that did not present photoprotective activity to the virus since it only caused approximately 0–8% mortality of *C*. *chalcites* post-UV exposure. This group consisted of kaolinite, benzopurpurine and benzimidazole. In the field assay, however, two UV-protective substances, charcoal and cacao, were selected based on their protective activity at cross-linker and their market price.

### UV protection efficacy of 1% cacao and 1% charcoal under field conditions

Cumulative radiation measurements (W/m^2^) were significantly higher (t = 3.535, df = 60, *P* = .001) in 2017 than in 2018 throughout the entire study period. The mean radiation values were 235.5 W/m^2^ and 181.6 W/m^2^ in 2017 and 2018, respectively. During the experiment in 2017, the average temperature was 23.81°C, the minimum temperature was 17.64°C and the maximum temperature was 28.21°C. The average temperature in 2018 was 18.41°C with a minimum of 17.55°C and a maximum of 26.08°C. The registered average humidity was 52.79% in 2017 and 77.67% in 2018 during the experiments.

**Fig 3:**
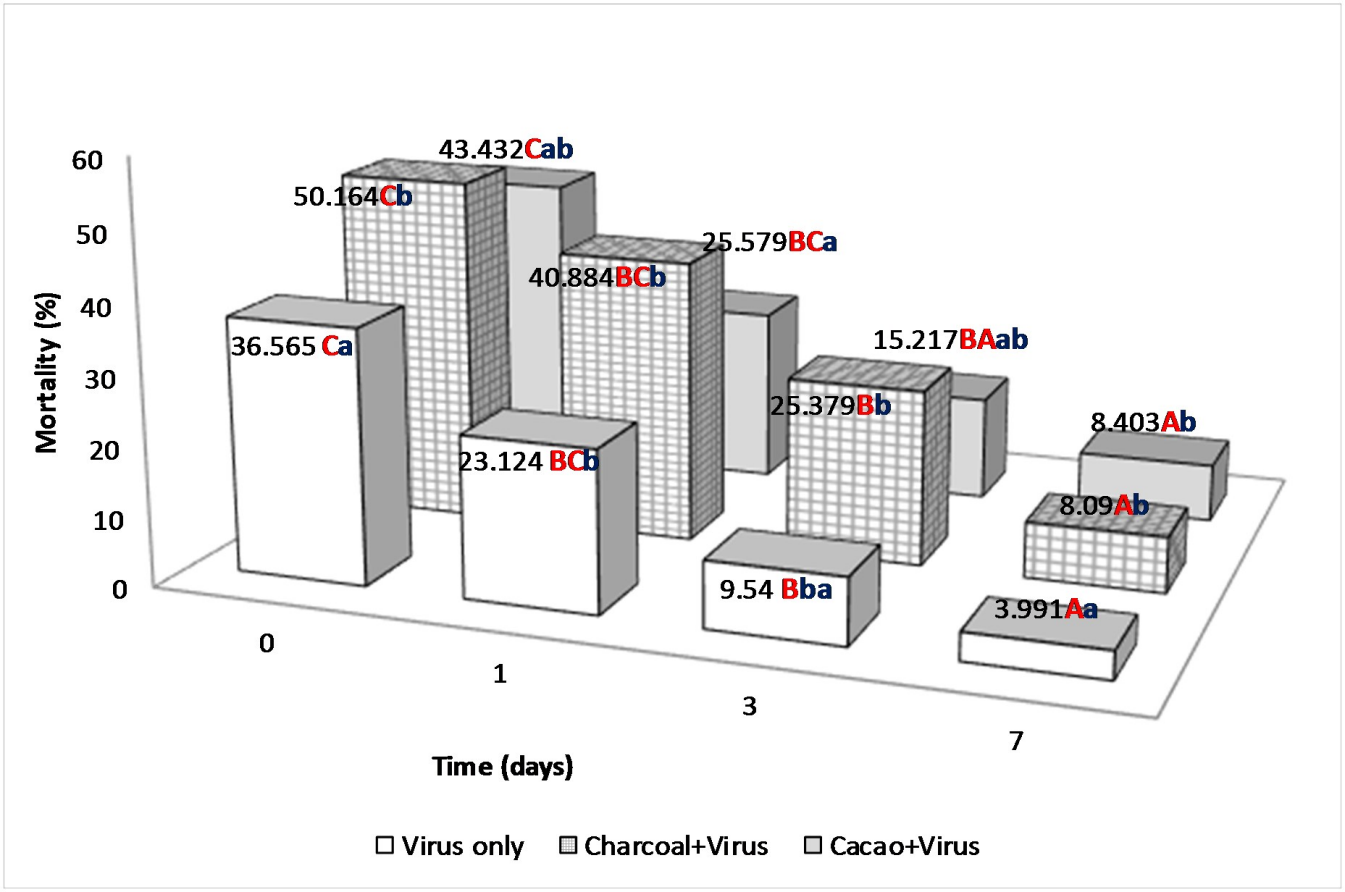
Percentage of mortality of 2nd instar larvae of *C*. *chalcites* at 0, 1, 3 and 7 days post application (2017). Treatments: (i) Charcoal 1% + ChchNPV-TF1, (ii) Cacao 1% + ChchNPV-TF1, (iii) Control (water + ChchNPV-TF1). *Capital letters represents differences among the treatments, lower case letters represent differences among the days post application.

**Fig 4:**
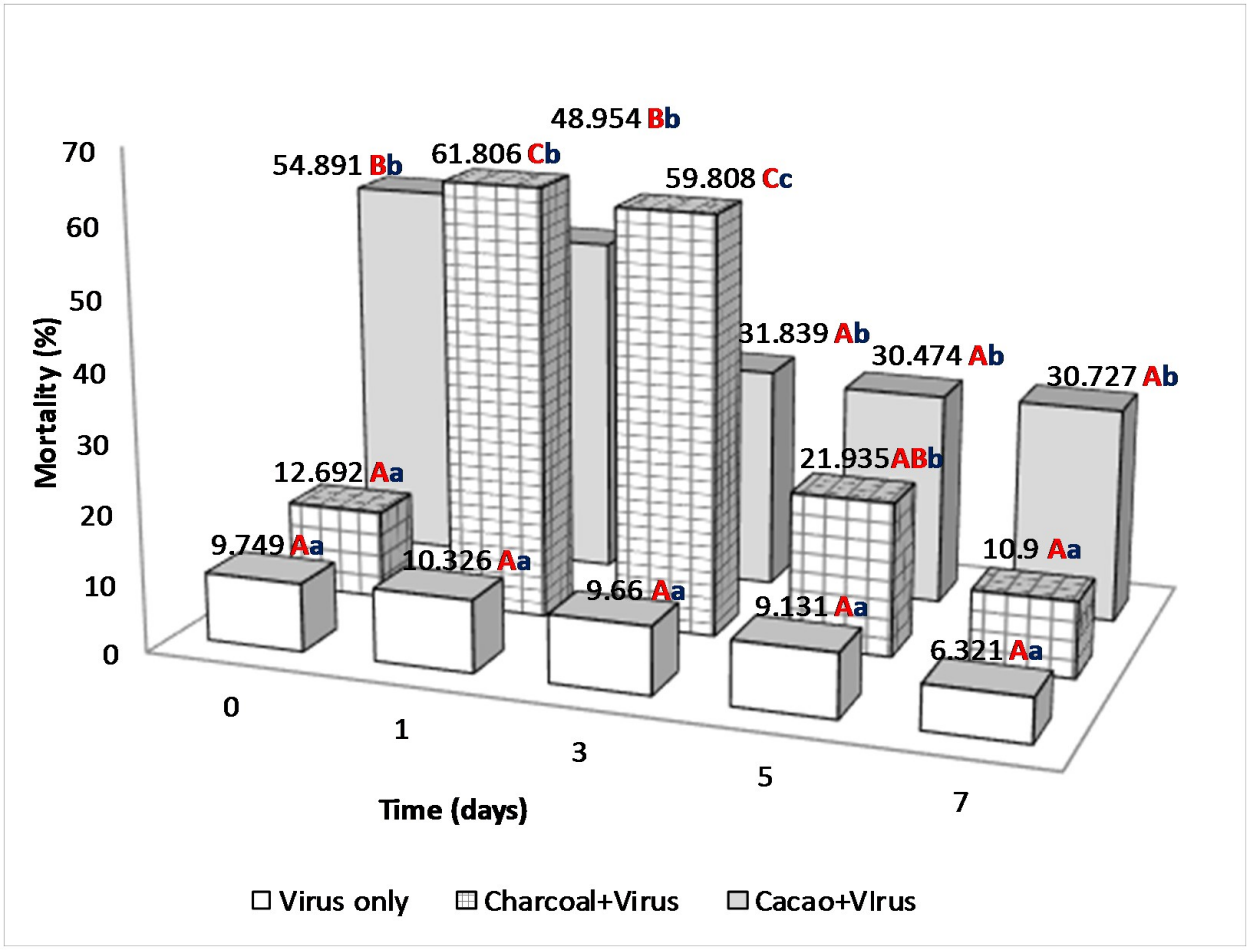
Percentage of mortality of 2nd instar larvae of *C*. *chalcites* at 0, 1, 3, 5 and 7 days post application (2018). Treatments: (i) Charcoal 1% + ChchNPV-TF1, (ii) Cacao 1% + ChchNPV-TF1, (iii) Control (water + ChchNPV-TF1). *Capital letters represents differences among the treatments, lower case letters represent differences among the days post application.

The percentages of mortality of 2^nd^ instar *C*. *chalcites* obtained in 2017 and 2018 are presented in Figs 3 and 4, respectively.

In the 2017 trial, mortality rates were significantly higher (F_2,28_ = 36.021, *P* < .0001) in charcoal + ChchNPV-TF1 (50.16 ± 10.79%) than in cacao + ChchNPV-TF1 treatment (43.43 ± 11.95%), although the mortality in cacao + ChchNPV-TF1 was not significantly greater than the mortality in control plants (ChchNPV-TF1 alone) (36.56 ± 7.62%). At 24 h postapplication, charcoal + ChchNPV-TF1 produced higher mortality rates (40.88 ± 9.44%) than cacao + ChchNPV-TF1 (25.57 ± 6.89%) (F_2,33_ = 29.959, *P* < .0001). Three days postapplication, significant differences were detected between treatments (F_2,33_ = 4.361, *P* = .047), charcoal + ChchNPV-TF1 (25.37 ± 5.44%), cacao + ChchNPV-TF1 (15.21 ± 2.32%), and control plants (9.53 ± 3.04%). Seven days postapplication, the mortality rates did not differ significantly (F_2.33_ = 0.862, *P* = 0.454) between treatments: cacao + ChchNPV-TF1 (8.40 ± 3.78%), charcoal + ChchNPV-TF1 (8.09 ± 1.71%) and control plants (ChchNPV-TF1 alone) (3.99 ± 1.94).

In the 2018 trial, the persistence of ChchNPV-TF1 varied significantly among postapplication days (F_4,50_ = 21.450, *P* < .0001) and among the four treatments (F_3,14_ = 17.869, *P* < .0001). The mortality rates after 1 h of exposure to UV were significantly higher (F_3,33_ = 44.613, *P* < .0001) in charcoal + ChchNPV-TF1 (54.89 ± 9.34%) than in cacao + ChchNPV-TF1 and control plants (9.74 ± 4.0%). After a 24-h period, the mortality of *C*. *chalcites* fed on plants treated with charcoal + ChchNPV-TF1 (61.80 ± 7.57%) was significantly higher (F_2,33_ = 29.959, *P* < .0001) than that of *C*. *chalcites* fed on plants treated with cacao + ChchNPV-TF1 (48.95 ± 12.19%) and on control plants (10.32 ± 3.11%). At three days postapplication, a similar tendency was observed. The mortality of *C*. *chalcites* was significantly higher in the cacao + ChchNPV-TF1 treatment (59.80 ± 5.91%) than in the charcoal + ChchNPV-TF1 (31.83 ± 9.27%) and control plants (9.66 ± 3.03%) (F_2,33_ = 43.178, *P* < .0001). Five days postapplication, mortality was significantly different between treatments, charcoal + ChchNPV-TF1 samples showed the higher percentage of mortality (30.47 ± 7.37%) followed by cacao + ChchNPV-TF1 (21.93 ± 6.32%) treatment and control plants (9.13 ± 2.5%) (F_2,33_ = 10.321, *P* < .0001). On the last day of sampling, *C*. *chalcites* mortality was significantly different (F_2,33_ = 12.089, *P* < .0001); the mortality rate remained at approximately 30.72% ± 9.86 on charcoal + ChchNPV-TF1 and 10.90 ± 4.34% on cacao+ChchNPV-TF1 and 6.32 ± 2.99% on control plants.

**Fig 5:**
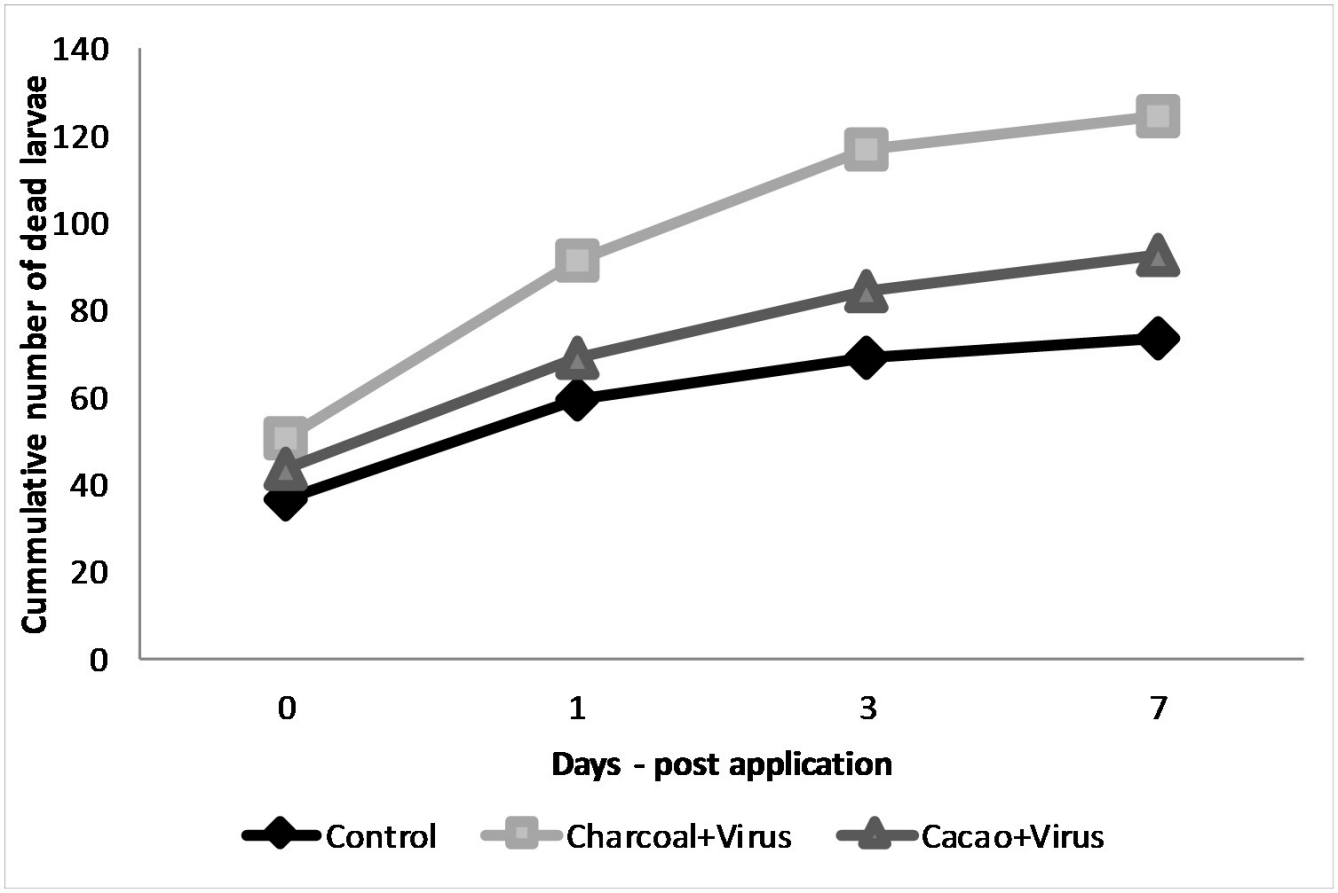
Cummulative larval mortality (2017).

**Fig 6:**
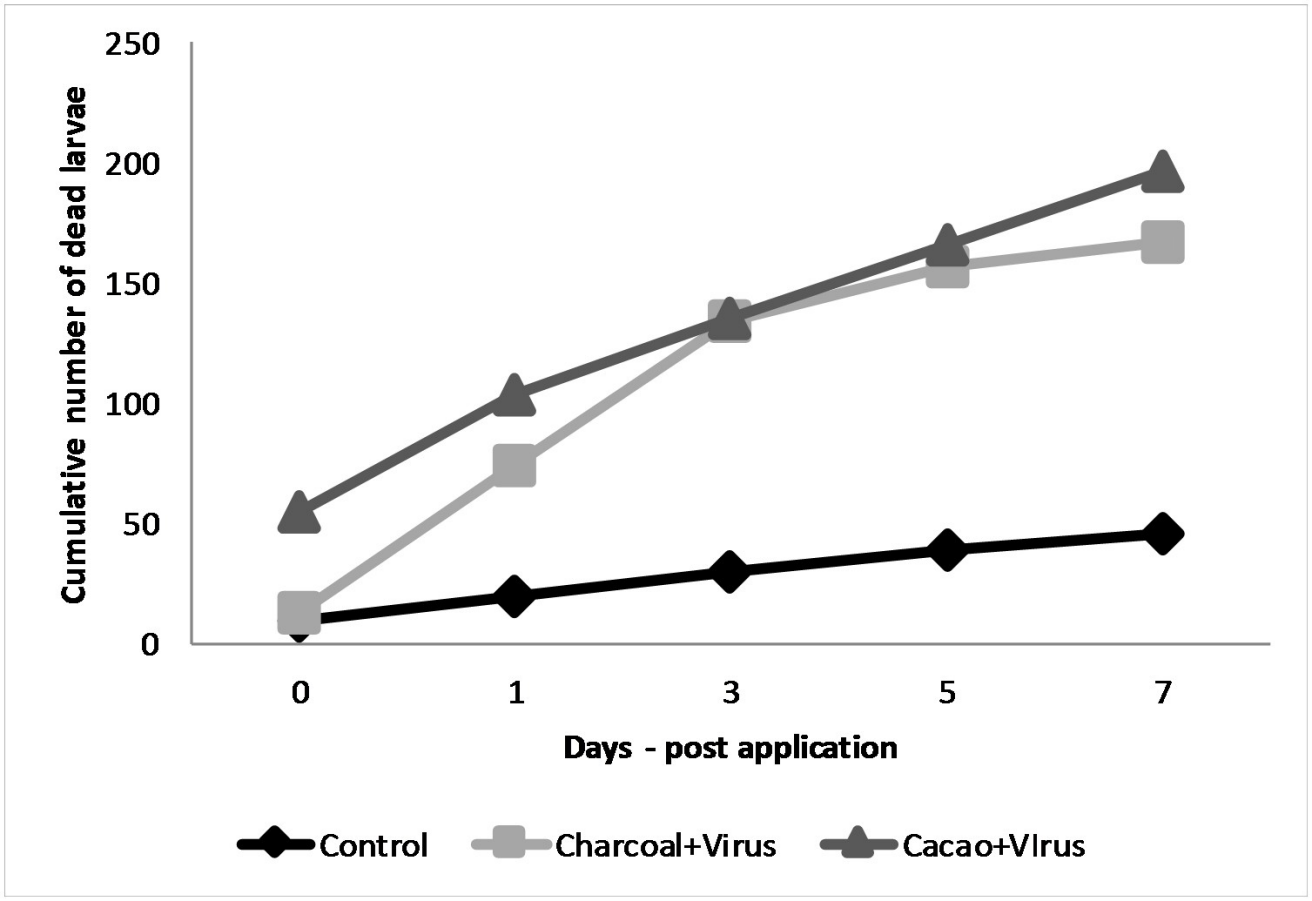
Cummulative larval mortality (2018).

Further data exploration demonstrated that in 2017 the cumulative baculovirus-induced mortality of *C*. *chalcites* was significantly higher in the charcoal + ChchNPV-TF1 treatment than in the cacao + ChchNPV-TF1 treatment at all sampling times (F_2,33_ = 3.044, *P* < .0001) (Fig 5). At all time points, control treatment mortality values were below UV-protective treatments. In 2018, cumulative virus-induced mortality was also significantly higher in the cacao+ChchNPV-TF1 treatment, followed by the charcoal + ChchNPV-TF1 treatment and, finally the control treatment for the first 2 time points (F_2,33_ = 55.399, *P* < .0001) (Fig 6). At all time points, control treatment mortality values were below UV-protective treatments.

### Influence of ChchSNPV-TF1 protectant substances on photosynthetic activity

No significant difference was found between the UV-protective substance-treated and control plants at 1, 3, 5, 7 and 14 days postapplication in 2018 (Table 2). The mean Fv/Fm values per treatment ranged from 0.74 for the water and ChchNPV-TF1 treatments and 0.75 for charcoal + ChchNPV-TF1 and cacao + ChchNPV-TF1.

**Table 2:** Maximum photochemical efficiency (Fv/Fm) values (Mean ± SD) between treatments after 1, 3, 5, 7 and 14 days after application in 2018 trial.

Treatments	Fv/Fm					
	0 day	1 day	3 days	5 days	7 days	14 days
Charcoal	0.76 ± 0.02	0.73 ± 0.03	0.72 ± 0.02	0.75 ± 0.01a	0.79 ± 0.02	0.74 ± 0.01
Cacao	0.71 ± 0.02	0.76 ± 0.01	0.74 ± 0.02	0.75 ± 0.01a	0.82 ± 0.01	0.74 ± 0.02
Control (-)	0.74 ± 0.01	0.78 ± 0.01	0.70 ± 0.03	0.72 ± 0.01b	0.76 ± 0.02	0.73 ± 0.02
Control (+)	0.72 ± 0.06	0.76 ± 0.01	0.71 ± 0.01	0.74 ± 0.01ab	0.78 ± 0.01	0.74 ± 0.02
*df*	3,6	3,35	3,35	3,43	3,44	3,44
*F*	0.254	1.326	0.510	3.236	1.860	0.064
*p*	0.856	0.282	0.678	0.031	0.150	0.979

Charcoal: charcoal 1%, Cacao: cacao 1%, Control (-): water only, Control (+): water + virus. Means in the same column with the same letter(s) are not significantly different (Tukey HSD, *p* < .05).

## Discussion

In this study, the efficacy of selected natural UV-protectant substances was evaluated under laboratory and field conditions as the basis for improving the formulation of the insecticide based on ChchNPV-TF1 to be employed in the biological control of *C*. *chalcites* in banana.

There is considerable evidence that solar radiation is one of the most important factors affecting the persistence of microbial insecticides on plants [13, 39, 41]. In particular, the ultraviolet (UV) portion of the spectrum (280–320 nm wavelength) is responsible for rapid inactivation of baculoviruses [42, 43]. In fact, when the efficacy of ChchNPV-TF1 was determined under greenhouse and open-field conditions in the Canary Islands, *C*. *chalcites* mortality observed under greenhouse conditions was fourfold higher than that observed under open-air conditions, indicating the need to improve baculovirus formulations for open–air applications [11].

The selection of natural additives in the present study was based on their previous use in laboratory or field experiments. In the first portion of our study, iron oxide showed high protection of ChchNPV-TF1 under laboratory conditions together with charcoal. However, we selected charcoal and cacao for field trials as they are readily available and less expensive than other protectants.

The application of both additives together with ChchNPV-TF1 resulted in an extended period of *C*. *chalcites* larval mortality. Those larvae exposed to the leaves collected at up to 7 days after treatment continued to show mortalities caused by lethal virus disease, while the same OB concentration used in 2007 trials without UV protectants showed a decrease in efficacy 24 h postapplication [10]. Our results indicate a greater persistence of ChchSNPV-TF1 OBs on the banana plant with respect to the application of virus alone, thereby suggesting that the addition of 1% charcoal or 1% cacao to ChchSNPV-TF1 10^9^ OBs/l solution may extend the period of pest control, producing larval mortality for longer periods by protecting OBs from UV degradation.

The persistence of ChchNPV-TF1 when 1% charcoal was added to the suspension was significantly higher than that when ChchNPV-TF1 was applied alone or 1% cacao+ChchNPV-TF1 in both years of field trials. Charcoal is a lightweight black carbon residue produced by removing water and other volatile constituents from animal and plant materials. These results are in agreement with those reported by several authors [12, 44], who discussed the photoprotective effect of charcoal with *Heliothis zea* nuclear polyhedrosis virus (HzSNPV).

Some authors have mentioned another effect of UV protectants that could be enhancing the baculovirus effect on the insect. These substances could act as a physiological stress factor, rendering the insect more susceptible to virus infection, probably by damaging the larva peritrophic membrane. This damaged membrane would allow a large number of virions to enter the ectoperitrophic space, invade susceptible cells and replicate [45, 46].

On the other hand, the protection offered by cacao was less effective, especially in the 2018 trial. Cacao has been studied as a UV protector for *Spodoptera littoralis* nucleopolyhedrovirus (*Spli*MNPV) [25] and for *Spodoptera exigua* nucleopolyhedrovirus (*Se*MNPV) [22]. The essential substance of cacao is theobromine, formerly known as xantheose, which is a bitter alkaloid of the cacao plant [47] that remains and colours the leaves when applied.

Such protective substances could have an effect on the photochemical efficacy of the treated plants, but no differences were observed in photochemical activity between the treatments and control plants during our 2018 experiments. This result implies that cacao and charcoal could be safe for use as UV absorbers for ChchNPV-TF1 in banana plantations, since they provide protection for the virus, thereby increasing its efficacy over time and not interfering with photosynthetic efficiency.

Banana plants have been frequently cultivated in regions with high radiation rates [48]. In both years, there was a high degradation in the number of viable OBs on direct UV light when no additive was used. Introducing these natural substances into the development of a commercial ChchNPV-Tf1 formulation would improve its persistence, since these substances have a significant effect on the number of viable OBs over time when exposed to direct sunlight. Moreover, considering that climate change is a phenomenon producing increased temperatures that may also increase the sensitivity of baculovirus OBs to UV radiation [48], the use of natural UV-protective substances appears to be of great importance when IPM needs to be established on the application of insecticides based on baculovirus, as is the case for *C*. *chalcites*.

This study provide promising results that these substances may be useful for the future biological control of *C*. *chalcites* with ChchSNPV-TF1 formulations. However, it is also necessary to compare greenhouse-based experiments and open-field trials and obtain results with a wider range of UV-protective substances under more varied conditions to develop a competitive commercial formulation for ChchSNPV-TF1.

## Supporting information

S1 Raw images (PDF)
