# Supplementary material for: Effects of several UV-protective substances on the persistence of the insecticidal activity of the Alphabaculovirus of Chrysodeixis chalcites (ChchNPV-TF1) on banana (Musa acuminata, Musaceae, Colla) under laboratory and open-field conditions
Source: PLoS One. 2021 May 12;16(5):e0250217. doi: 10.1371/journal.pone.0250217 (PMC8115783; doi:10.1371/journal.pone.0250217)
Supplement: S1 Raw images — (PDF) [file pone.0250217.s001.pdf]

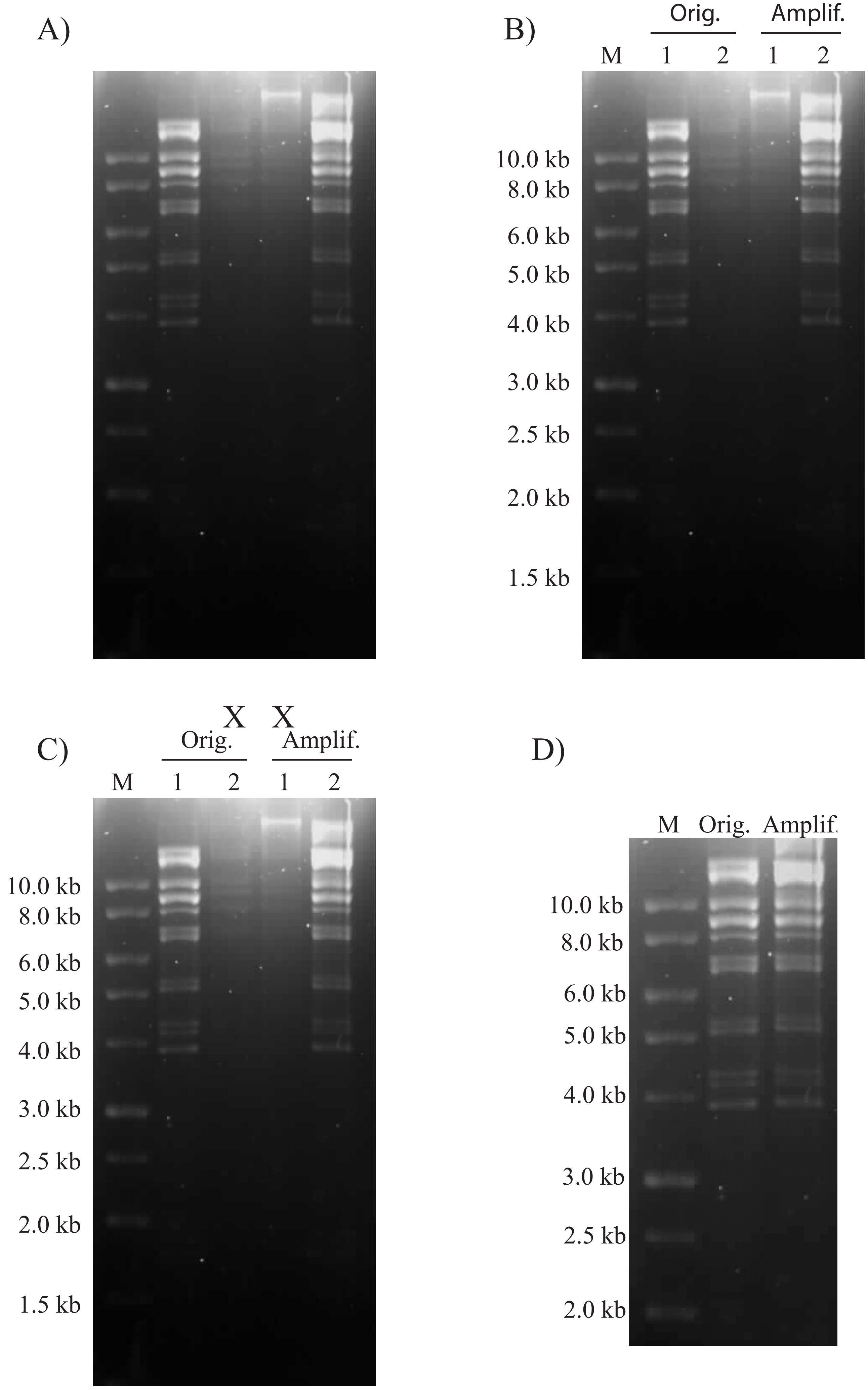

S1_raw_images. A) Original picture of the restriction profiles taken in a UV Transilluminator (Chemi-Doc, BioRad, California, USA) of the original ChchNPV-TF1 isolate and that massively produced or amplified in larvae. Two DNA extractions (two replicas) were performed por each sample. In each sample one of the DNA extractions failed or were not visible, probably due to low DNA quantity or a failure during DNA extraction. B) Annotated original picture. Line 1 (M) Molecular marker HypperLadder 1Kb (Bioline), molecular weights of the fragments are indicated on the left. Line 2 (Orig. 1) Restriction profiles of the original ChchNPV-TF1 replica 1. Line 3 (Orig. 2) Restriction profiles of the original ChchNPV-TF1 replica 2. Line 4 (Amplif. 1) Restriction profiles of the massively produced (amplified) ChchNPV-TF1 in larva replica 1. Line 5 (Orig. 2) Restriction profiles of the massively produced (amplified) ChchNPV-TF1 in larva replica 2. C) Annotated picture indicating the lines cropped or removed (indicated with a X above the lines). D) Final figure with lines 3 and 4 cropped.
